# Supplementary material for: Mismatch between media coverage and research on invasive species: The case of wild boar (Sus scrofa) in Argentina
Source: PLoS One. 2022 Dec 22;17(12):e0279601. doi: 10.1371/journal.pone.0279601 (PMC9778503; doi:10.1371/journal.pone.0279601)
Supplement: S1 Table — (DOCX) [file pone.0279601.s001.docx]

S1 Table

Links to the news websites used in this study about wild boar in Argentina published until 2020 (N=194), indicating the year of publication and the scope of news coverage (provincial or national).

| **Link** | **Year** | **Location** |
| --- | --- | --- |
| <https://www.diario26.com/55446--jabali-se-come-un-caballo-y-atemoriza-a-14-personas> | 2007 | Mendoza |
| <https://www.region.com.ar/caza/nuevo-record-nacional-de-jabali-julian-querejeta-974.html> | 2010 | La Pampa |
| <https://www.region.com.ar/caza/resultados-cuarto-torneo-de-caza-de-jabali-con-jauria-2010-960.html> | 2010 | La Pampa |
| <https://www.region.com.ar/caza/resultados-fotos-torneo-jabali-jauria-dogo-cuchillo-2010-953.html> | 2010 | La Pampa |
| <https://www.region.com.ar/caza/cazador-logra-trofeo-de-jabali-4-en-el-ranking-959.html> | 2010 | La Pampa |
| <https://www.region.com.ar/caza/resultados-torneo-jabali-asociacion-nores-martinez-1002.html> | 2011 | La Pampa |
| <https://diariojornada.com.ar/51451/Politica/Ademas_de_los_jabalies_preocupa_el_avance_de_pumas_en_la_meseta> | 2012 | Chubut |
| <https://www.ellitoral.com.ar/corrientes/2012-12-19-1-0-0-productores-preocupados-por-los-cerdos-salvajes> | 2012 | Corrientes |
| <https://www.diariouno.com.ar/mendoza/se-desato-una-caceria-sistematica-de-jabalies-en-tunuyan-para-detener-una-creciente-plaga-invasiva-05122012_r1Lyo2fzBX> | 2012 | Mendoza |
| <https://www.rionegro.com.ar/brote-de-triquinosis-afecto-a-30-personas-en-GRRN_1148163/> | 2013 | Neuquén |
| <https://www.eleco.com.ar/interes-general/instan-a-cazadores-de-jabalies-y-a-quienes-realizan-carneadas-a-realizar-analisis-de-triquina/> | 2014 | Buenos Aires |
| <https://www.region.com.ar/productos/semanario/archivo/noticias-la-pampa/torneo-caza-jabali-quehue-resultados-1195.html> | 2015 | La Pampa |
| <https://www.region.com.ar/caza/resultados-torneo-jabali-asociacion-nores-martinez-1002.html> | 2015 | La Pampa |
| <https://www.diariojornada.com.ar/116692/sociedad/Un_jabali_mordio_a_un_turista_en_Bariloche> | 2015 | Río Negro |
| <http://www.corrientesnoticias.com.ar/noticias/view/159223> | 2016 | Corrientes |
| <https://www.diarioelargentino.com.ar/noticias/169248/Autorizan-cazar-el-ciervo-axis-y-el-jabali-durante-todo-el-ano-en-toda-la-provincia> | 2016 | Entre Ríos |
| <https://www.region.com.ar/productos/semanario/archivo/noticias-la-pampa/11er-torneo-de-caza-de-jabali-1224.html> | 2016 | La Pampa |
| <https://www.region.com.ar/productos/semanario/archivo/noticias-la-pampa/torneo-de-caza-de-jabali-al-acecho-en-quehue-1221.html> | 2016 | La Pampa |
| <https://www.losandes.com.ar/article/atraparon-a-tres-cazadores-furtivos-con-un-jabali-muerto-y-armas-en-tunuyan> | 2016 | Mendoza |
| <https://www.lmneuquen.com/por-esquivar-un-jabali-perdio-el-control-y-volco-la-ruta-40-n517511> | 2016 | Neuquén |
| <https://www.rionegro.com.ar/el-jabali-una-amenaza-para-la-produccion-zonal-BM1860663/> | 2016 | Río Negro |
| <https://www.adnrionegro.com.ar/2016/03/un-auto-choco-contra-un-chancho-jabali-tres-heridos/> | 2016 | Río Negro |
| <https://www.adnrionegro.com.ar/2016/03/una-investigacion-de-la-unrn-alerta-sobre-posibles-enfermedades-que-puede-transmitir-el-jabali/> | 2016 | Río Negro |
| <https://www.adnrionegro.com.ar/2016/12/el-jabali-es-un-riesgo-sanitario-para-el-campo-por-brucelosis-y-tuberculosis/> | 2016 | Río Negro |
| <http://notinogo.blogspot.com/2015/05/preocupacion-por-plaga-de-jabalies-en.html> | 2016 | San Luis |
| <http://www.radiobelgranosuardi.com.ar/noticiasinterior.php?id=4118> | 2016 | Santa Fé |
| <http://appnoticias.com.ar/app/en-patagones-flexibilizan-la-caza-de-jabali-y-comenzaron-las-reuniones-con-productores-ganaderos-para-prevenir-incendios-de-campos/> | 2017 | Buenos Aires |
| <https://www.lanacion.com.ar/sociedad/en-carmen-de-patagones-autorizan-cazar-jabalies-porque-son-una-plaga-nid2046330> | 2017 | Buenos Aires |
| <https://www.noticiasnet.com.ar/118-noticiasnet/patagones/interes-general-patagones/38480-el-chancho-jabali-es-una-plaga-terrible> | 2017 | Buenos Aires |
| <https://www.noticiasnet.com.ar/118-noticiasnet/patagones/interes-general-patagones/40088-caza-de-chancho-jabali-una-vieja-practica-en-la-region> | 2017 | Buenos Aires |
| <https://zonalpress.com.ar/nota/51/en-carmen-de-patagones-autorizan-cazar-jabalies-porque-son-una-plaga> | 2017 | Buenos Aires |
| <https://www.diariojornada.com.ar/186089/policiales/secuestran_carne_de_guanaco_y_jabali/> | 2017 | Chubut |
| <https://www.diariojornada.com.ar/186089/policiales/secuestran_carne_de_guanaco_y_jabali/> | 2017 | Chubut |
| <https://www.lavoz.com.ar/ciudadanos/en-cordoba-se-pueden-cazar-jabalies-ciervos-bufalos-y-antilopes> | 2017 | Córdoba |
| <https://www.telam.com.ar/notas/201701/176463-corrientes-yaguarete-paraguay.html> | 2017 | Corrientes |
| <http://archivo.laarena.com.ar/la_ciudad-jabalies-cambian-de-habitos-ahora-matan-ovejas-1152137-115.html> | 2017 | La Pampa |
| <https://www.diariojornada.com.ar/199658/sociedad/cientificos_del_conicet_estudian_el_impacto_del_jabali_y_el_conejo_europeo_dos_especies_invasoras/> | 2017 | Mendoza |
| <https://www.rionegro.com.ar/el-jabali-avanza-sin-control-y-ahora-arrasa-con-las-pasturas-en-pulmari-EK3518116/> | 2017 | Neuquén |
| <https://www.rionegro.com.ar/detectan-23-casos-de-triquinosis-en-jabalies-de-la-cordillera-IX2132695/> | 2017 | Neuquén |
| <http://appnoticias.com.ar/app/a-diferencia-de-otras-jurisdicciones-en-rio-negro-se-permite-la-caza-deportiva-y-de-control-del-jabali-todo-el-ano/> | 2017 | Río Negro |
| <https://www.rionegro.com.ar/proponen-incorporar-la-carne-de-jabali-a-la-gastronomia-rionegrina-NJ2920852/> | 2017 | Río Negro |
| <http://appnoticias.com.ar/app/patagones-autorizaron-cazar-jabalies-con-jaurias-desde-2019/> | 2018 | Buenos Aires |
| <https://www.ciudadanodiario.com.ar/nota/2018-5-23-10-45-2-exquisito-costillar-de-jabali-a-la-parrilla> | 2018 | Buenos Aires |
| <https://www.eleco.com.ar/la-ciudad/aparecieron-casos-positivos-de-triquinosis-en-dos-cerdos-de-la-zona-que-ya-fueron-faenados/> | 2018 | Buenos Aires |
| <https://www.noticiasnet.com.ar/48252-trabajan-para-reglamentar-la-caza-del-chancho-jabali> | 2018 | Buenos Aires |
| <https://www.noticiasnet.com.ar/113-noticiasnet/patagones/politica-patagones/51712-se-aprobo-la-caza-de-chancho-jabali-con-perros> | 2018 | Buenos Aires |
| <https://www.noticiasnet.com.ar/118-noticiasnet/patagones/interes-general-patagones/49603-caza-del-chancho-jabali-se-viene-la-reglamentacion> | 2018 | Buenos Aires |
| <https://www.noticiasnet.com.ar/113-noticiasnet/patagones/politica-patagones/51255-proponen-prohibir-la-caza-de-jabali-con-perros> | 2018 | Buenos Aires |
| <https://tn.com.ar/tecno/twittendencias/un-cerdo-entro-una-universidad-y-la-foto-genero-una-batalla-de-memes_875841> | 2018 | Buenos Aires |
| <https://vos.lavoz.com.ar/comer-y-beber/jabali-al-disco-en-nueva-cordoba-nuevo-cafe-italiano-y-mas> | 2018 | Córdoba |
| <http://www.laarena.com.ar/la_pampa-que-paso-con-el-jabali-suelto-en-santa-rosa-2027570-163.html> | 2018 | La Pampa |
| https://diariosanrafael.com.ar/las-unicas-especies-permitidas-para-la-caza-son-el-jabali-y-la-liebre-de-castilla-75249/ | 2018 | Mendoza |
| <https://www.losandes.com.ar/article/view?slug=desbarataron-a-un-cazador-en-san-rafael-tenia-la-cabeza-de-un-jabali-arriba-del-techo> | 2018 | Mendoza |
| <https://ruralaldia.com/noticias/actualidad/nacional/3603/encuestan-a-productores-por-las-perdidas-que-genera-el-jabali/> | 2018 | National |
| <https://news.agrofy.com.ar/noticia/174390/encuesta-productores-estimar-impacto-cerdo-silvestre-argentina> | 2018 | National |
| <https://www.anbariloche.com.ar/noticias/2018/09/14/65923-jabalies-mataron-salvajemente-a-un-perro-en-el-este> | 2018 | Río Negro |
| <https://www.elcordillerano.com.ar/noticias/2018/11/12/72932-mito-y-realidad-del-avance-delos-jabalies-en-zonas-urbanas-> | 2018 | Río Negro |
| <http://www.revista-airelibre.com/2018/09/18/jabalies-en-bariloche/> | 2018 | Río Negro |
| <https://www.rionegro.com.ar/recomendaciones-ante-la-posible-faena-domesticas-de-jabali-FB5322430/> | 2018 | Río Negro |
| <https://www.anbariloche.com.ar/noticias/2018/09/19/65974-una-gran-invasion-los-jabalies-se-encuentran-en-el-70por_ciento-de-la-superficie-del-parque-nacional-nahuel-huapi> | 2018 | Río Negro |
| <https://www.clarin.com/sociedad/bariloche-alerta-jabalies-merodean-ciudad_0_SJ7jPU6um.html> | 2018 | Río Negro |
| <https://www.anbariloche.com.ar/noticias/2018/09/18/65959-como-llegaron-los-jabalies-a-bariloche-y-por-que-se-convirtieron-en-una-plaga> | 2018 | Río Negro |
| <https://www.anbariloche.com.ar/noticias/2018/09/20/65991-que-medidas-se-deben-tomar-para-controlar-la-poblacion-del-jabali-en-bariloche> | 2018 | Río Negro |
| <https://www.rionegro.com.ar/atraparon-un-jabali-en-costa-del-sol-AM5709662/> | 2018 | Río Negro |
| <https://www.rionegro.com.ar/chorizos-de-jabali-en-auge-pero-con-las-prevenciones-YH5669166/> | 2018 | Río Negro |
| <https://www.red43.com.ar/nota/2018-8-12-20-40-0-choco-en-la-ruta-40-contra-un-chancho-jabali> | 2018 | Río Negro |
| <https://www.rionegro.com.ar/chorizos-de-jabali-en-auge-pero-con-las-prevenciones-YH5669166/> | 2018 | Río Negro |
| <https://www.telam.com.ar/notas/201810/298536-caza-jabali-san-luis.html> | 2018 | San Luis |
| <http://cadenadenoticias.com.ar/otro-accidente-de-transito-con-un-chancho-jabali-en-una-autopista-de-la-provincia/> | 2018 | San Luis |
| <http://cadenadenoticias.com.ar/salvaron-sus-vidas-de-milagro-tras-impactar-con-una-manada-de-chanchos-jabalies/> | 2018 | San Luis |
| <https://www.radiodigitalsanluis.com/post/un-jabal%C3%AD-provoc%C3%B3-un-accidente-en-la-autopista-55> | 2018 | San Luis |
| <https://www.eldiariodelarepublica.com/nota/2018-4-3-10-53-0-un-chancho-jabali-se-cruzo-en-la-ruta-y-provoco-un-accidente-automovilistico> | 2018 | San Luis |
| <https://www.abcsaladillo.com.ar/saladillo/informacion-general/preocupacion-en-el-campo-por-la-presencia-de-jabalies-en-el-centro-de-la-provincia/> | 2019 | Buenos Aires |
| <https://news.agrofy.com.ar/noticia/181337/buscan-reducir-poblacion-cerdos-silvestres-territorio-bonaerense> | 2019 | Buenos Aires |
| <http://www.elpopular.com.ar/134609> | 2019 | Buenos Aires |
| <http://www.elpopular.com.ar/134935> | 2019 | Buenos Aires |
| <https://dib.com.ar/2019/04/autorizan-caza-de-jabali-en-distritos-para-evitar-danos-en-produccion-agropecuaria/> | 2019 | Buenos Aires |
| <https://www.eleco.com.ar/la-ciudad/esta-habilitada-la-caceria-en-tandil/> | 2019 | Buenos Aires |
| <https://www.elfederal.com.ar/buscan-reducir-la-poblacion-de-cerdos-silvestres-y-proteger-al-venado-de-las-pampas/> | 2019 | Buenos Aires |
| <https://enlineanoticias.com.ar/secciones/ciudad/caza-de-jabali-en-campos-de-olavarria-fotos-al-100/> | 2019 | Buenos Aires |
| <https://enlineanoticias.com.ar/secciones/ciudad/preocupa-la-aparicion-de-jabalies-en-campos-de-olavarria/> | 2019 | Buenos Aires |
| <https://www.infocampo.com.ar/autorizan-la-caza-plaguicida-de-jabali-en-la-provincia-de-buenos-aires/> | 2019 | Buenos Aires |
| <https://www.lanacion.com.ar/sociedad/carnes-no-tradicionales-un-nicho-que-crece-de-la-mano-de-la-cocina-gourmet-nid2239516> | 2019 | Buenos Aires |
| <https://lavozdelpueblo.com.ar/noticia/86343-Preocupaci%C3%B3n-en-el-campo-por-la-presencia-de-jabal%C3%ADes-en-el-centro-de-la-provincia> | 2019 | Buenos Aires |
| <https://www.noticiasnet.com.ar/nota/2019-6-27-10-7-0-caza-de-jabali-y-preocupacion-de-productores> | 2019 | Buenos Aires |
| <https://www.noticiasnet.com.ar/nota/2019-7-8-8-22-0--son-los-propios-perros-de-los-duenos-los-que-hacen-dano> | 2019 | Buenos Aires |
| <https://www.noticiasnet.com.ar/nota/2019-5-14-9-40-0-con-algunas-demoras-burocraticas-se-desarrolla-la-caza-del-jabali> | 2019 | Buenos Aires |
| <https://www.noticiasnet.com.ar/nota/2019-6-13-14-10-0-fuga-y-aprehendidos-con-dos-chanchos-jabali> | 2019 | Buenos Aires |
| <https://www.noticiasnet.com.ar/nota/2019-7-4-8-44-0-caza-de-jabali-por-un-lado-cazadores-y-por-otro-los-delincuentes> | 2019 | Buenos Aires |
| <http://www.noticiasradioreflejos.com.ar/noticias/index.php/2019/05/23/un-vecinos-de-sierra-de-la-ventana-choco-contra-un-jabali-en-la-zona-de-7-puentes-de-la-ruta-51/> | 2019 | Buenos Aires |
| <https://www.ohlaprida.com.ar/2019/04/azul-los-detienen-con-perros-y-una-chancha-jabali-muerta/> | 2019 | Buenos Aires |
| <https://www.revistachacra.com.ar/nota/14451-ajustan-controles-en-patagones-ante-la-nueva-plaga-el-jabali/> | 2019 | Buenos Aires |
| <https://www.semanarioextra.com.ar/jabalies-sueltos-preocupacion-en-la-provincia-de-buenos-aires/> | 2019 | Buenos Aires |
| <https://www.telam.com.ar/notas/201911/412947-comarca-sierras-de-la-ventana-turismo-verano.html> | 2019 | Buenos Aires |
| <https://www.zonacampo.com.ar/locales-y-regionales/desde-bromatologia-advierten-sobre-el-consumo-de-carne-de-jabali> | 2019 | Buenos Aires |
| <https://www.zonacampo.com.ar/infogen/preocupa-la-aparicion-de-jabalies-en-campos-de-olavarria> | 2019 | Buenos Aires |
| <https://www.diariojornada.com.ar/252605/sociedad/festival_comodoro_invita_y_sus_especialidades_gastronomicas/> | 2019 | Chubut |
| <https://agroverdad.com.ar/2019/05/impulsaran-la-industria-carnica-del-jabali-para-mitigar-la-sobrepoblacion-en-cordoba> | 2019 | Córdoba |
| <https://elchorrillero.com/nota/2019/04/21/139716-un-automovilista-esquivo-un-jabali-y-termino-contra-una-arboleda/amp/> | 2019 | Córdoba |
| <https://www.eldiariodecarlospaz.com.ar/provincial/2019/4/17/ambiente-busca-controlar-al-jabali-europeo-en-cordoba-66717.html> | 2019 | Córdoba |
| <http://laola.com.ar/preocupacion-por-manadas-de-jabalies-en-calamuchita/> | 2019 | Córdoba |
| <https://www.lavoz.com.ar/ciudadanos/secuestraron-96-aves-y-un-jabali-en-santa-rosa-de-calamuchita> | 2019 | Córdoba |
| https://www.lavoz.com.ar/ciudadanos/jabalies-se-puede-cazar-en-casi-toda-provincia-pero-solo-de-noche | 2019 | Córdoba |
| <https://www.lavoz.com.ar/ciudadanos/en-sierras-hay-cada-vez-mas-chanchos-cimarrones> | 2019 | Córdoba |
| <https://www.lavoz.com.ar/sucesos/chocaron-un-jabali-auto-quedo-destruido-chancho-terminara-en-parrilla> | 2019 | Córdoba |
| <https://vos.lavoz.com.ar/comer-y-beber/cartof-una-de-las-aperturas-del-ano-para-la-gastronomia-cordobesa> | 2019 | Córdoba |
| <https://www.ellitoral.com.ar/corrientes/2019-10-15-1-19-0-decomisaron-armas-para-caza-y-animales-silvestres-en-localidades-del-interior> | 2019 | Corrientes |
| <http://diariolarepublica.com.ar/notix/noticia/10953/alertan-sobre-daos-que-causan-los-chanchos-salvajes-en-el-ecosistema-y-la-produccin-.html> | 2019 | Corrientes |
| <https://www.diarioriouruguay.com.ar/policiales/un-joven-fue-atacado-por-chancho-salvaje-cuando-intentaba-cazarlo.htm> | 2019 | Entre Ríos |
| <https://www.primeraedicion.com.ar/nota/100144962/el-gobierno-ratifica-autorizacion-para-cazar-dos-especies-en-el-palmar/> | 2019 | Entre Ríos |
| <https://federalaldia.com.ar/policiales/chanar-un-chancho-salvaje-provoco-graves-heridas-a-un-trabajador-rural/> | 2019 | Entre Ríos |
| <http://www.nogoyaaldia.com.ar/index.php/locales/4767-por-esquivar-un-chancho-salvaje-un-auto-es-envestido-de-atras-por-una-camioneta-cerca-de-lucas-gonzalez> | 2019 | Entre Ríos |
| <https://www.unoentrerios.com.ar/policiales/mataron-un-carpincho-y-llevaban-dos-crias-jabali-n2508499.html> | 2019 | Entre Ríos |
| <https://www.argentina.gob.ar/noticias/la-pampa-se-evito-el-consumo-de-carne-de-dos-jabalies-con-triquinosis> | 2019 | La Pampa |
| <https://www.enbocadetodoshd.com.ar/mi-barrio/2019/1/14/barrio-federal-vecinos-denunciaron-que-hay-un-chancho-salvaje-recorriendo-las-calles-mira-las-fotos-31542.html> | 2019 | La Pampa |
| <https://infotecrealico.com.ar/contenido/11440/pincen-colisionaron-con-un-jabali-en-ruta-26> | 2019 | La Pampa |
| <http://www.laarena.com.ar/la_pampa-sorprenden-a-furtivos-2-2056795-163.html> | 2019 | La Pampa |
| <http://www.laarena.com.ar/la_pampa-un-jabali-hizo-volvar-un-auto-con-dos-funcionarios-2086448-163.html> | 2019 | La Pampa |
| <http://www.laarena.com.ar/la_pampa-extienden-periodo-de-habilitacion-para-la-caza-deportiva-de-jabali-y-antilope-2072146-163.html> | 2019 | La Pampa |
| <http://www.laarena.com.ar/la_arena_del_campo-una-actividad-ilegal-que-crece-2090106-16.html> | 2019 | La Pampa |
| <http://www.laarena.com.ar/la_pampa-sorprenden-a-un-furtivo-castense-2085658-163.html> | 2019 | La Pampa |
| <http://www.laarena.com.ar/la_pampa-tenian-un-jabali-como-mascota-2044151-163.html> | 2019 | La Pampa |
| <http://www.laarena.com.ar/la_pampa-demoraron-a-ocho-cazadores-2079862-163.html> | 2019 | La Pampa |
| <http://www.laarena.com.ar/la_pampa-sorprenden-a-furtivos-2-2056795-163.html> | 2019 | La Pampa |
| <http://www.laarena.com.ar/la_pampa-tenian-un-jabali-como-mascota-2044151-163.html> | 2019 | La Pampa |
| <http://www.lapostadesanluis.com.ar/2019/06/choque-entre-un-automovil-y-un-chancho.html> | 2019 | La Pampa |
| <https://www.region.com.ar/productos/semanario/archivo/noticias-la-pampa/quehue-fiesta-caza-2019-1385.html> | 2019 | La Pampa |
| <https://www.region.com.ar/productos/semanario/archivo/noticias-la-pampa/extienden-temporada-caza-jabali-antilope-2019-1376.html> | 2019 | La Pampa |
| <https://www.region.com.ar/productos/semanario/archivo/noticias-la-pampa/torneo-caza-jabali-jauria-acecho-2019-1374.html> | 2019 | La Pampa |
| <https://www.region.com.ar/caza/nuevo-record-nacional-de-jabali-julian-querejeta-974.html> | 2019 | La Pampa |
| <https://www.losandes.com.ar/article/view?slug=comio-carne-de-jabali-y-contrajo-triquinosis> | 2019 | Mendoza |
| <https://www.losandes.com.ar/article/view?slug=para-controlar-especies-invasoras-el-gobierno-habilito-cotos-de-caza> | 2019 | Mendoza |
| <https://www.losandes.com.ar/article/view?slug=permiten-la-caza-de-cuatro-animales-considerados-daninos> | 2019 | Mendoza |
| <https://www.infocampo.com.ar/el-gobierno-de-mendoza-autorizo-la-caza-deportiva-de-cuatro-especies-exoticas/> | 2019 | Mendoza |
| <https://www.losandes.com.ar/article/view?slug=permiten-la-caza-de-algunas-especies-de-conejos-jabalies-y-otros-animales-en-mendoza> | 2019 | Mendoza |
| <https://www.lanacion.com.ar/sociedad/controversia-por-la-habilitacion-de-caza-de-especies-exoticas-en-mendoza-nid2300351> | 2019 | Mendoza |
| <https://realidadsm.com/2019/01/10/una-pareja-que-viajaba-en-moto-terminaron-internados-al-embestir-a-un-chancho-que-se-les-cruzo-en-la-ruta/> | 2019 | Neuquén |
| <https://www.lmneuquen.com/se-le-cruzo-un-chancho-cuando-viajaba-su-moto-la-ruta-40-y-termino-el-hospital-n619455> | 2019 | Neuquén |
| <https://www.rionegro.com.ar/jabalies-las-estrategias-para-controlar-la-poblacion-en-nahuel-huapi-1166651/> | 2019 | Río Negro |
| <https://www.lanacion.com.ar/sociedad/bariloche-preocupa-la-invasion-jabalies-crece-temor-nid2303732> | 2019 | Río Negro |
| <https://www.rionegro.com.ar/este-domingo-a-valle-azul-como-hacer-encurtidos-de-cebolla-y-pera-escabeches-de-liebre-y-chorizos-hidratados-en-cerveza-1186017/> | 2019 | Río Negro |
| <https://www.rionegro.com.ar/con-diversas-propuestas-guardia-mitre-celebra-sus-157-anos-1202014/> | 2019 | Río Negro |
| <http://appnoticias.com.ar/app/con-gran-exito-se-desarrollo-el-1-concurso-de-jabali-a-la-estaca-en-guardia-mitre/> | 2019 | Río Negro |
| <https://www.anbariloche.com.ar/noticias/2019/09/09/71223-se-le-cruzo-un-chancho-en-la-ruta-y-volco> | 2019 | Río Negro |
| <https://www.rionegro.com.ar/jabalies-las-estrategias-para-controlar-la-poblacion-en-nahuel-huapi-1166651/> | 2019 | Río Negro |
| <https://www.rionegro.com.ar/la-poblacion-de-jabalies-crece-y-preocupa-a-bariloche-1161600/> | 2019 | Río Negro |
| <https://www.losandes.com.ar/article/view?slug=iban-a-hacerse-dialisis-y-murieron-al-chocar-contra-un-jabali-en-san-luis> | 2019 | San Luis |
| <https://viapais.com.ar/san-luis/1359897-fotografiaron-a-un-enorme-jabali-en-san-luis/> | 2019 | San Luis |
| <https://cuyonoticias.com/100856/san-luis-un-chancho-jabali-ocasiono-un-tremendo-choque-y-dos-hombres-murieron/> | 2019 | San Luis |
| <http://www.periodistasenlared.info/julio19-04/nota11.html> | 2019 | San Luis |
| <https://www.radiopopularsanluis.com.ar/policiales/2019/6/24/la-jefa-de-la-comisaria-de-juan-llerena-choco-un-chancho-jabali-37177.html> | 2019 | San Luis |
| <https://viapais.com.ar/santa-fe/957832-alertan-sobre-chanchos-salvajes-que-destrozan-campos-y-atacan-a-la-fauna/> | 2019 | Santa Fé |
| <https://www.eldia.com/nota/2020-6-8-2-43-57-jabalies-la-plaga-que-amenaza-a-mas-de-media-provincia-informacion-general> | 2020 | Buenos Aires |
| <https://www.eleco.com.ar/la-ciudad/desde-bromatologia-recordaron-que-el-rotulo-libre-de-triquinosis-es-garantia-de-inocuidad/> | 2020 | Buenos Aires |
| <https://www.agritotal.com/nota/42723-un-jabali-siembra-el-terror-en-localidades-de-9-de-julio/> | 2020 | Buenos Aires |
| <https://www.infozona.com.ar/afirman-que-los-jabalies-se-volvieron-plaga-y-estan-fuera-de-control/> | 2020 | Buenos Aires |
| <https://www.lanacion.com.ar/lifestyle/delivery-sabores-patrios-locros-empanadas-mas-barrios-nid2360071> | 2020 | Buenos Aires |
| <https://www.lanueva.com/nota/2020-6-20-6-30-48-el-chancho-jabali-como-pez-en-el-agua-en-el-sur-del-sudoeste-bonaerense> | 2020 | Buenos Aires |
| <https://www.noticiasnet.com.ar/nota/2020-5-13-14-1-0-cazadores-de-chancho-piden-autorizacion-para-cazar> | 2020 | Buenos Aires |
| <https://www.noticiasnet.com.ar/nota/2020-3-11-8-42-0-habilitaran-la-caza-plaguicida-de-jabali> | 2020 | Buenos Aires |
| <https://saltoenred.com.ar/bromatologia-informa-a-quienes-elaboren-chacinados/> | 2020 | Buenos Aires |
| <https://weekend.perfil.com/noticias/caza/sur-de-buenos-aires-jabalies-plaga-descontrol.phtml> | 2020 | Buenos Aires |
| <https://www.elchubut.com.ar/nota/2020-5-9-19-38-0--un-jabali-en-la-doble-trocha> | 2020 | Chubut |
| <https://lu17.com/tambien-es-noticia/advierten-por-el-avance-del-jabali-en-campos-de-peninsula> | 2020 | Chubut |
| <https://laopinionaustral.com.ar/informacion-general/peninsula-valdes-fue-a-pescar-y-lo-sorprendio-un-jabali-158101.html> | 2020 | Chubut |
| <https://www.lanueva.com/nota/2020-4-21-11-25-0-una-familia-resulto-ilesa-tras-chocar-con-el-auto-a-un-jabali> | 2020 | Córdoba |
| <https://vos.lavoz.com.ar/comer-y-beber/cuarentena-nuevos-restaurantes-al-delivery> | 2020 | Córdoba |
| <https://viapais.com.ar/carlos-paz/1743052-accidente-en-las-altas-cumbres-un-automovil-atropello-a-un-jabali/> | 2020 | Córdoba |
| <https://www.maximaonline.com.ar/Nota-59138-seis_detenidos_por_violar_la_cuarentena_y_la_ley_de_caza> | 2020 | Corrientes |
| <https://www.diarioelargentino.com.ar/noticias/204934/cazadores-furtivos-en-plena-cuarentena> | 2020 | Entre Ríos |
| <http://www.apfdigital.com.ar/despachos.asp?cod_des=337979&ID_Seccion=3&fecemi=20/04/2020&Titular=detuvieron-a-seis-cazadores-furtivos-en-plena-cuarentena-en-entre-riacuteos.html> | 2020 | Entre Ríos |
| <https://entreriosya.com.ar/los-detuvieron-con-un-animal-porcino-cruza-con-jabali-en-la-caja-de-su-camioneta-en-federal/> | 2020 | Entre Ríos |
| <https://infohuella.com.ar/contenido/8760/luan-toro-cazan-como-furtivos-publican-en-redes-sociales-y-terminan-detenidos> | 2020 | La Pampa |
| <https://infohuella.com.ar/contenido/8728/luan-toro-allanamiento-tres-hermanos-detenidos-y-liberacion-de-animales-silvestr> | 2020 | La Pampa |
| <https://www.infocampo.com.ar/confirmaron-un-nuevo-caso-de-triquinosis-en-la-pampa/> | 2020 | La Pampa |
| <http://www.laarena.com.ar/la_pampa-demoraron-a-cinco-cazadores-2112866-163.html> | 2020 | La Pampa |
| <http://www.laarena.com.ar/la_pampa-demoran-a-cazadores-3-2114058-163.html> | 2020 | La Pampa |
| <http://www.laarena.com.ar/la_pampa-secuestraron-mas-de-100-kg-de-carne-de-jabali-con-triquinosis-en-general-acha-2092944-163.html> | 2020 | La Pampa |
| <http://www.laarena.com.ar/la_pampa-loventue-dos-hombres-detenidos-por-caza-ilegal-2108869-163.html> | 2020 | La Pampa |
| <http://www.laarena.com.ar/la_pampa-caleufu-sorprenden-a-un-furtivo-2109610-163.html> | 2020 | La Pampa |
| <http://www.laarena.com.ar/la_pampa-luan-toro-detienen-a-dos-cazadores-furtivos-2107650-163.html> | 2020 | La Pampa |
| <http://www.laarena.com.ar/la_pampa-sorprenden-a-cazador-2111155-163.html> | 2020 | La Pampa |
| <https://www.elcordillerano.com.ar/noticias/2020/06/29/92235-alertan-sobre-el-avance-del-jabali-en-los-parques-nacionales> | 2020 | Neuquén |
| <https://www.noticiasnet.com.ar/nota/2020-5-30-12-49-0-alertan-sobre-los-riesgos-de-la-triquinosis> | 2020 | Río Negro |
| <https://www.diariodecuyo.com.ar/sanjuan/La-proliferacion-de-jabalies-complica-a-productores-vallistos-y-buscan-que-se-los-declare-plaga-20200519-0064.html> | 2020 | San Juan |
| <https://www.diariodecuyo.com.ar/sanjuan/Estiman-que-en-2-meses-resolveran-si-es-plaga-el-jabali-y-eventualmente-como-reducirlo-20200520-0091.html> | 2020 | San Juan |
| https://www.noticiasnet.com.ar/nota/2020-10-22-7-19-0-sin-permiso-provincial-igualmente-se-caza-jabali-en-la-region | 2020 | Buenos Aires |
| https://www.noticiasnet.com.ar/nota/2020-12-4-7-28-0-podrian-habilitar-la-caza-de-jabali-durante-todo-el-verano | 2020 | Buenos Aires |
| https://www.noticiasnet.com.ar/nota/2020-12-21-17-52-0-choco-contra-un-jabali-en-la-ruta-que-une-viedma-con-el-condor | 2020 | Río Negro |
| https://diariolaopinion.com.ar/contenido/293128/rafaelino-demorado-con-armas-y-carne-de-jabali | 2020 | Santa Fé |
| https://corrientesinfo.com.ar/una-nueva-ley-habilita-a-reducir-la-poblacion-de-chanchos-salvajes-en-corrientes/ | 2020 | Corrientes |
| https://corrientesinfo.com.ar/una-nueva-ley-habilita-a-reducir-la-poblacion-de-chanchos-salvajes-en-corrientes/ | 2020 | Santa Fé |
| https://www.noticiasnet.com.ar/nota/2020-12-24-12-46-0-el-jabali-transformar-una-especie-invasora-en-una-oportunidad-de-negocio | 2020 | Río Negro |
| https://www.elcordillerano.com.ar/noticias/2020/11/21/99171-tres-hombres-fueron-demorados-por-cazar-jabalies | 2020 | Río Negro |
