# Supplementary material for: Mismatch between media coverage and research on invasive species: The case of wild boar (Sus scrofa) in Argentina
Source: PLoS One. 2022 Dec 22;17(12):e0279601. doi: 10.1371/journal.pone.0279601 (PMC9778503; doi:10.1371/journal.pone.0279601)
Supplement: S2 Table — (DOCX) [file pone.0279601.s002.docx]

S2 Table

Scientific articles published on wild boar in Argentina published until 2020 (N=37), indicating the authors, title, journal, year of publication and the regional scope (provincial or national).

| Authors | Title | Year | Journal | Location |
| --- | --- | --- | --- | --- |
| Merino ML, Carpinetti B N | Feral pig *Sus scrofa* population estimates in Bahía Samborombón conservation area, Buenos Aires province, Argentina | 2003 | Mastozoología Neotropical | Buenos Aires |
| Vieites CM, Basso CP, Bartolini N | Wild boar (*Sus scrofa ferus*): Productivity index in an experimental outdoor farm. | 2003 | InVet | Buenos Aires |
| Pérez Carusi LC, Beade MS, Miñarro F, Vila A R, Giménez-Dixon M, Bilenca DN | Relaciones espaciales y numéricas entre venados de las pampas (*Ozotoceros bezoarticus celer*) y chanchos cimarrones (*Sus scrofa*) en el Refugio de Vida Silvestre Bahía Samborombón, Argentina. | 2009 | Ecología Austral | Buenos Aires |
| Pescador M, Sanguinetti J, Pastore H, Peris S | Expansion of the introduced wild boar (*Sus scrofa*) in the Andean region, Argentinean Patagonia | 2009 | Galemys | Neuquén, Rio Negro, Chubut |
| Cohen M, Costantino SN, Calcagno MA, Blanco GA, Pozio E, Venturiello SM | Trichinella infection in wild boars (*Sus scrofa*) from a protected area of Argentina and its relationship with the presence of humans | 2010 | Veterinary Parasitology | Entre Ríos |
| Cuevas MF, Novillo A, Campos C, Dacar MA, Ojeda RA | Food habits and impact of rooting behaviour of the invasive wild boar, *Sus scrofa*, in a protected area of the Monte Desert, Argentina | 2010 | Journal of Arid Environments | Mendoza |
| Sanguinetti J, Kitzberger T | Factors controlling seed predation by rodents and non-native *Sus scrofa* in Araucaria araucana forests: potential effects on seedling establishment | 2010 | Biological Invasions | Neuquén |
| Meikle V, Bianco MV, Blanco FC, Gioffre A, Garbaccio S, Vagnoni L, et al. | Evaluation of pathogenesis caused in cattle and guinea pig by a *Mycobacterium bovis* strain isolated from wild boar | 2011 | Bmc Veterinary Research | Buenos Aires, La pampa y Santa Fe |
| Cuevas MF, Mastrantonio L, Ojeda RA, Jaksic FM | Effects of wild boar disturbance on vegetation and soil properties in the Monte Desert, Argentina | 2012 | Mammalian Biology | Mendoza |
| Schiaffini MI, Vila AR | Habitat use of the wild boar, *Sus scrofa* Linnaeus 1758, in Los Alerces National Park, Argentina | 2012 | Studies on Neotropical Fauna and Environment | Chubut |
| Cuevas MF, Ojeda RA, Dacar MA, Jaksic FM | Seasonal variation in feeding habits and diet selection by wild boars in a semi-arid environment of Argentina | 2013 | Acta Theriologica | Mendoza |
| Cuevas MF, Ojeda RA, Jaksic FM | Multi-scale patterns of habitat use by wild boar in the Monte Desert of Argentina | 2013 | Basic and Applied Ecology | Mendoza |
| Barrios‐Garcia MN, Simberloff D | Linking the pattern to the mechanism: How an introduced mammal facilitates plant invasions | 2013 | Austral Ecology | Neuquén |
| Barrios-Garcia MN, Classen AT, Simberloff D | Disparate responses of above- and belowground properties to soil disturbance by an invasive mammal | 2014 | Ecosphere | Neuquén |
| Ballari SA, Cuevas MF, Cirignoli S, Valenzuela AEJ | Invasive wild boar in Argentina: using protected areas as a research platform to determine distribution, impacts and management | 2015 | Biological Invasions | Argentina |
| Ballari SA, Cuevas MF, Ojeda RA, Navarro JL | Diet of wild boar (*Sus scrofa*) in a protected area of Argentina: the importance of baiting | 2015 | Mammal Research | Entre Ríos |
| Gurtler RE, Izquierdo VM, Gil G, Cavicchia M, Maranta A | Coping with wild boar in a conservation area: impacts of a 10-year management control program in north-eastern Argentina | 2016 | Biological Invasions | Entre Ríos |
| Martinez JIZ, Santillan MA, Sarasola JH, Travaini A | A native top predator relies on exotic prey inside a protected area: The puma and the introduced ungulates in Central Argentina | 2016 | Journal of Arid Environments | La Pampa |
| Carpinetti B, Di Guirolamo G, Delgado JV, Martínez RD | El cerdo criollo costero: valioso recurso zoogenético local de la provincia de Buenos Aires Argentina. | 2016 | Archivos de Zootecnia, | Buenos Aires |
| Cuevas MF, Ojeda RA, Jaksic Andrade FM | Ecological strategies and impact of wild boar in phytogeographic provinces of Argentina with emphasis on aridlands. | 2016 | Mastozoología Neotropical | Argentina |
| Sanguinetti J, Pastore H | Abundancia poblacional y manejo del jabalí (*Sus scrofa*): una revisión global para abordar su gestión en la Argentina. | 2016 | Mastozoología Neotropical | Argentina |
| Carusi LCP, Beade MS, Bilenca DN | Spatial segregation among pampas deer and exotic ungulates: a comparative analysis at site and landscape scales | 2017 | Journal of Mammalogy | Buenos Aires |
| Gurtler RE Rodriguez-Planes LI, Gil G, Izquierdo VM, Cavicchia M, Maranta A | Differential long-term impacts of a management control program of axis deer and wild boar in a protected area of north-eastern Argentina | 2017 | Biological Invasions | Entre Ríos |
| Soteras F, Ibarra C, Geml J, Barrios-Garcia MN, Dominguez LS, Nouhra ER | Mycophagy by invasive wild boar (*Sus scrofa*) facilitates dispersal of native and introduced mycorrhizal fungi in Patagonia, Argentina | 2017 | Fungal Ecology | Neuquén |
| Carpinetti B, Castresana G, Rojas P, Grant J, Marcos A, Monterubbianesi M, et al. | Determinación de anticuerpos contra patógenos virales y bacterianos seleccionados en la población de cerdos silvestres (*Sus scrofa*) de la Reserva Natural Bahía Samborombón Argentina. | 2017 | Analecta Veterinaria | Buenos Aires |
| Caruso N, Valenzuela AEJ, Burdett CL, Vidal EML, Birochio D, Casanave EB | Summer habitat use and activity patterns of wild boar *Sus scrofa* in rangelands of central Argentina | 2018 | Plos One | Buenos Aires |
| Sagua MI, Figueroa CE, Acosta DB, Fernandez GP, Carpinetti BN, Birochio D, et al. | Inferring the origin and genetic diversity of the introduced wild boar (*Sus scrofa*) populations in Argentina: an approach from mitochondrial markers | 2018 | Mammal Research | Argentina |
| Brizzio R, Alvarez M, Thern E, Daffner J | Caso clínico de pseudorrabia en canino de caza de jabalí (*Sus scrofa*) en el Valle Medio de Río Negro, Argentina. | 2018 | Revista de Medicina Veterinaria (Buenos Aires) | Río Negro |
| Acosta DB, Figueroa CE, Fernandez GP, Carpinetti BN, Merino ML | Genetic diversity and phylogenetic relationships in feral pig populations from Argentina | 2019 | Mammalian Biology | Argentina |
| Acosta DB, Ruiz M, Sanchez JP | First molecular detection of Mycoplasma suis in the pig louse *Haematopinus suis* (Phthiraptera: Anoplura) from Argentina | 2019 | Acta Tropica | Buenos Aires |
| Ballari SA, Hendrix BD, Sample M, Nunez MA | Management of invasive Pinaceae is imperiled by the lack of invasive ungulate control: successful restoration requires multiple-species management | 2019 | Mammal Research | Río Negro |
| Ciocco RB, Carpinetti BN, Rojas P, Castresana G, Notarnicola J | Endoparasites in a wild boar population (*Sus scrofa*) from Bahia Samborombon, Buenos Aires, Argentina | 2019 | Revista Mexicana de Biodiversidad | Buenos Aires |
| Pisano MB, Winter M, Raimondo N, Martinez-Wassaf MG, Abate SD, et al. | New pieces in the transmission cycle of the hepatitis E virus in South America: first viral detection in wild boars from Argentina | 2019 | Transactions of The Royal Society of Tropical Medicine and Hygiene | Rio Negro, Buenos Aires |
| Winter M, Abate SD, Pasqualetti MI, Farina FA, Ercole ME, Pardini L, et al. | *Toxoplasma gondii* and Trichinella infections in wild boars (*Sus scrofa*) from Northeastern Patagonia, Argentina | 2019 | Preventive Veterinary Medicine | Rio Negro, Buenos Aires |
| Panebianco A, Bó RF, Gregorio PF, Vila A | Macro and microhabitat patterns of habitat use and selection by wild boar in Los Alerces National Park | 2019 | Mastozoología Neotropical | Chubut |
| Ballari SA, Valenzuela AEJ, Nuñez MA | Interactions between wild boar and cattle in Patagonian temperate forest: cattle impacts are worse when alone than with wild boar | 2020 | Biological Invasions | Río Negro |
| Cuevas MF, Campos CM, Ojeda RA, Jaksic FM | Vegetation recovery after 11 years of wild boar exclusion in the Monte Desert, Argentina | 2020 | Biological Invasions | Mendoza |
